# Discharge Instruction Reminders Via Text Messages After Benign Gynecologic Surgery: Quasi-Experimental Feasibility Study

**DOI:** 10.2196/22681

**Published:** 2021-12-14

**Authors:** Jocelyn Sajnani, Kimberly Swan, Sharon Wolff, Kelsi Drummond

**Affiliations:** 1 University of Kansas School of Medicine University of Kansas Medical Center Kansas City, KS United States; 2 Department of Obstetrics and Gynecology University of California, Los Angeles Los Angeles, CA United States; 3 Department of Obstetrics and Gynecology Overland Park Regional Medical Center Overland Park, KS United States; 4 Department of Population Health University of Kansas Medical Center Kansas City, KS United States; 5 Department of Obstetrics and Gynecology University of Kansas Medical Center Kansas City, KS United States

**Keywords:** communication, hysterectomy, minimally invasive, laparoscopy, postoperative, patient satisfaction

## Abstract

**Background:**

With the implementation of enhanced recovery after surgery protocols and same-day hospital discharge, patients are required to take on increasing responsibility for their postoperative care. Various approaches to patient information delivery have been investigated and have demonstrated improvement in patient retention of instructions and patient satisfaction.

**Objective:**

This study aimed to evaluate the feasibility of implementing a postoperative text messaging service in the benign gynecologic population.

**Methods:**

We used a quasi-experimental study design to evaluate patients undergoing outpatient laparoscopic surgery for benign disease with a minimally invasive gynecologist at an academic medical center between October 2017 and March 2018. In addition to routine postoperative instructions, 19 text messages were designed to provide education and support to postoperative gynecologic patients. Patients were contacted by telephone 3 weeks postoperatively and surveyed about their satisfaction and feelings of connectedness during their recovery experience. Demographic and operative information was gathered through chart review. The cost to implement text messages was US $2.85 per patient.

**Results:**

A total of 185 patients were eligible to be included in this study. Of the 100 intended intervention participants, 20 failed to receive text messages, leaving an 80% success in text delivery. No patients opted out of messaging. A total of 28 patients did not participate in the postrecovery survey, leaving 137 patients with outcome data (control, n=75; texting, n=62). Satisfaction, determined by a score ≥9 on a 10-point scale, was 74% (46/62) in the texting group and 63% (47/75) in the control group (*P*=.15). Connectedness (score ≥9) was reported by 64% (40/62) in the texting group compared with 44% (33/75) in the control group (*P*=.02). Overall, 65% (40/62) of those in the texting group found the texts valuable (score ≥9).

**Conclusions:**

Postoperative text messages increased patients’ perceptions of connection with their health care team and may also increase their satisfaction with their recovery process. Errors in message delivery were identified. Given the increasing emphasis on patient experience and cost effectiveness in health care, an adequately powered future study to determine statistically significant differences in patient experience and resource use would be appropriate.

## Introduction

With the increase in same-day hospital discharge, patients are required to take on increasing responsibility for their postoperative care. Low health literacy has been consistently associated with poor health [1]. Adequate health literacy is required to follow discharge instructions and has a significant impact on the care of surgical patients [2]. Yet, despite patient education prior to discharge, patients continue to have questions about routine postoperative care when at home [3]. This may represent patient difficulty in retaining medical information, as patients tend to focus more on information related to their diagnosis at the expense of instructions regarding treatment [4]. The lack of information retention and associated poor outcomes underline the importance of continued improvement in patient education regarding discharge care.

Various approaches to patient information delivery have been investigated and have demonstrated improvement in patient retention of instruction. Successful studies have used pictographs [5], multimedia video [6], and other electronic reminders like text messages [7]. Text messaging may be particularly beneficial for the postoperative surgical population who have unique medical needs and require robust education about their postoperative care. A previous feasibility study examining patients undergoing breast reconstruction and anterior cruciate ligament repair found implementing a mobile application for monitoring quality of recovery at home was feasible and acceptable to patients [8].

Previous research can be leveraged and applied to the benign gynecologic postsurgical population through carefully curated text messages that provide education and support during the postoperative recovery period. The intent of this study was to evaluate the feasibility of implementing a postoperative text messaging service.

## Methods

### Sample and Messaging

**Figure 1:**
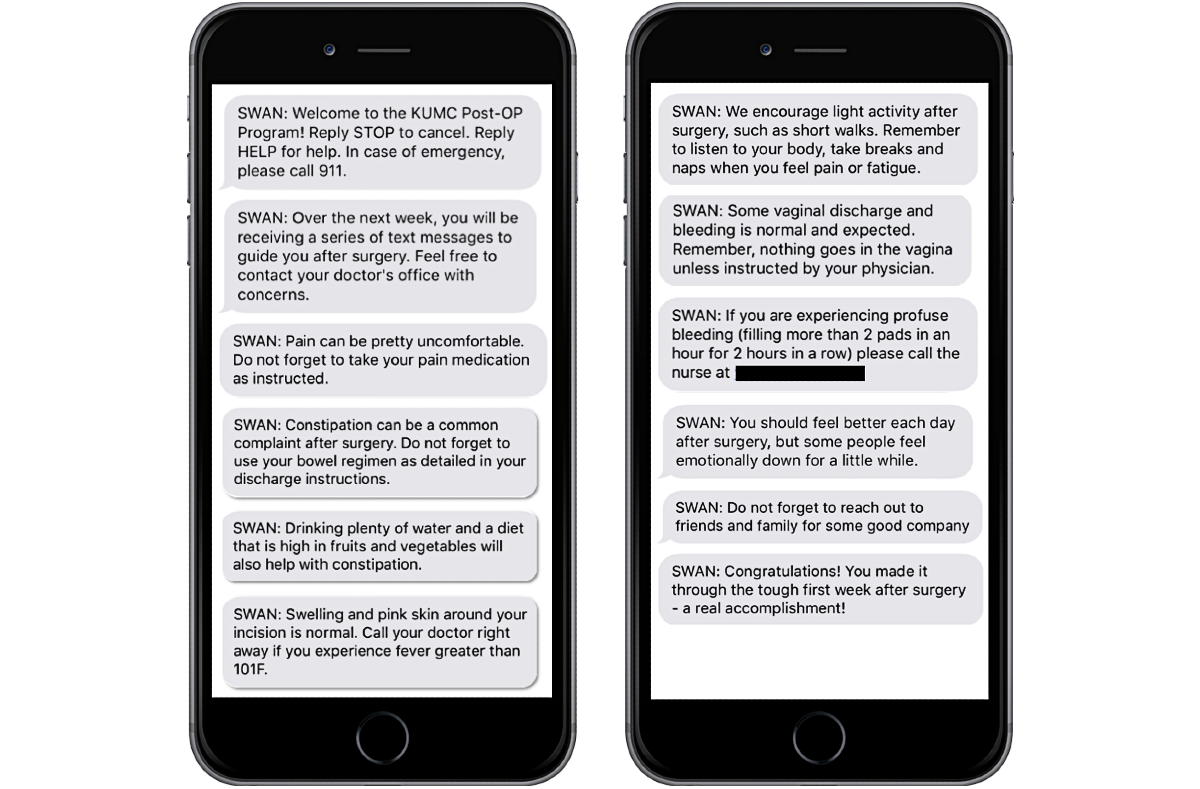
Selection of text messages delivered to the intervention group.

This quasi-experimental study included women undergoing benign gynecologic, laparoscopic surgery with a single minimally invasive gynecologist at an academic medical center over a 6-month period. The institutional review board approved this project with quality improvement determination. Patients were eligible for inclusion if they were at least 18 years old and their primary language was English. Those patients undergoing open procedures or laparoscopic procedures converted to open were excluded. For a 3-month period, the control group received routine discharge instructions after their procedures. During the subsequent 3-month period, the intervention group received routine discharge instructions in addition to text messages on days 1 through 8 following their procedure. These messages were implemented using an existing commercially available service for appointment reminder text messaging via TeleVox Solutions. Phone numbers for patients meeting inclusion criteria were pulled directly from patients’ charts. Initial attempt at messaging was made with listed mobile phone numbers. If the number was missing or inaccurate, a second attempt was made using listed home phone numbers, with the understanding that home phone numbers are often mobile numbers. In total, 19 text messages were transmitted to patients via one-way messaging, using their procedure date as an anchoring point. The messages were written to address common postoperative milestones, provide recovery tips, identify situations in which to escalate care, and lastly, to provide encouragement during the recovery process (Figure 1). Patients were universally opted into automated messaging, however, could elect to opt out upon receipt of the initial text message. The cost to implement text messaging was US $0.15 per text, which was the rate negotiated with TeleVox Solutions, with a total cost of US $2.85 per patient for the complete text series. Departmental research funds were used to cover the cost of messaging. Patients in both the control and texting groups were contacted 3 weeks postoperatively for participation in a phone survey. They were asked to rate the following on a scale of 1 to 10: (1) “How satisfied are you with your postoperative care?” (2) “How connected did you feel to your health care team while recovering at home?” (3) “How valuable did you find the text messages while recovering at home?”

The third question was only posed to the texting group. Additionally, demographic, procedural, and postoperative complication data were collected through chart review.

### Outcome Measures

As a pilot study, the primary intent was to determine the feasibility of implementing a text messaging protocol and successfully sending messages to patients. The primary endpoint was the percent of intended intervention patients who received all messages without error. Establishing that the intervention did not harm and might benefit patient care was necessary to justify continued evaluation of this intervention. Therefore, important secondary endpoints were patient scores on satisfaction, feelings of connection, and value of text messaging during their postoperative recovery. Satisfaction, connection, and value of text messaging were collected using a 10-point scale. These scores were collapsed into binary variables with scores of 9 and 10 coded as being satisfied, connected, and finding value. Scores of 1 through 8 represented a neutral or negative response. This determination was largely based on clinical importance.

### Statistical Analysis

Descriptive statistics were calculated for all outcome and clinical variables. The primary outcome was calculated as the proportion of patients who received all text messages out of those patients who were intended to receive messaging. Chi-squared analyses were used to compare patient characteristics as well as the outcomes of satisfaction and connectedness between the control and intervention groups. To determine if age modified the relationship between texting and the outcomes of satisfaction and connectedness, a Breslow-Day test was conducted. The median age of the cohort (37 years) was used to define younger (<37 years) and older (≥37 years) subgroups.

## Results

### Text Message Delivery Success Rate

**Figure 2:**
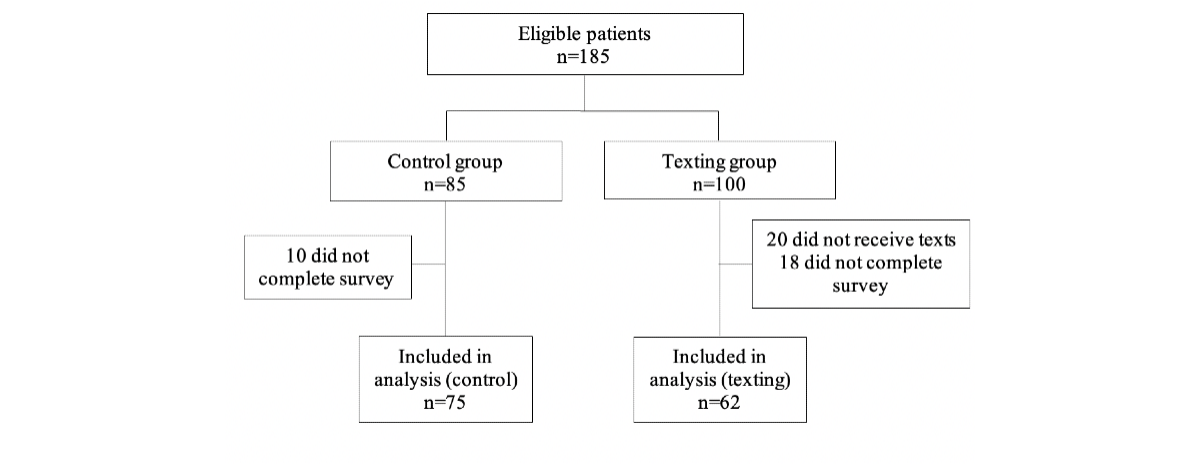
Flow diagram of study recruitment.

A total of 185 patients were identified to be included in this study. In the texting group, 20 patients did not receive the initial text message due to an error, likely due to the use of a landline phone number. In addition, 10 control and 18 intervention patients did not participate in the postrecovery survey, leaving 137 patients with outcome data (control, n=75; texting, n=62). The primary endpoint was the percent of intended intervention patients who received all messages without error. Of the 100 intended texting group patients, 20 did not receive messages due to error, leaving 80% (80/100) of the intended intervention patients successfully receiving all 19 text messages. Of note, no patients receiving messaging opted out after receiving the initial text, which included the choice to opt out (Figure 2).

### Participant Demographic and Clinical Data

The age of study participants ranged from 20 years to 59 years, with a mean age of 38.1 years (SD 8.8). Control and intervention patients were statistically different in terms of age (*P*=.04), with the mean age of the texting cohort being 2.9 years younger than that of the control group. Otherwise, the groups did not differ significantly in terms of other procedural or clinical metrics (Table 1).

**Table 1:** Demographic and clinical characteristics of the control and intervention groups.

Characteristic	Control group (n=75)	Texting group (n=62)	*P* value
Age (years), mean (SD)	39.5 (8.6)	36.4 (8.7)	.04
Overnight stays, n (%)	21 (28)	11 (18)	.16
Emergency department visit, n (%)	4 (5)	4 (7)	.78
Hysterectomy, n (%)	37 (49)	24 (39)	.21

### Patient Satisfaction With Postoperative Recovery

Based on completed survey responses, 63% (47/75) of patients receiving standard discharge instructions were satisfied with their postoperative care, while 74% (46/62) of patients receiving additional educational text messaging were satisfied (*P*=.15). Although the texting group patients were found to be younger, age did not modify patient-reported satisfaction scores. Patients <37 years old in the texting group rated their satisfaction similar to those ≥37 years old in the texting group (75%, 47/62 vs 73%, 55/75; *P*=.86). Type of operative procedure, categorized as hysterectomy or uterine-sparing, did play a role in patient satisfaction. For those in the control group with standard written instructions, 76% (28/37) of those patients undergoing hysterectomy were satisfied with their postoperative care, while only 50% (19/38) undergoing uterine-sparing procedures were satisfied (*P*=.02). In contrast, this relationship between type of procedure and satisfaction did not exist in the texting group (*P*=.91).

### Patient Connection With the Health Care Team

Survey responses showed that 64% (40/62) of patients receiving educational and supportive text messages felt more connected with their health care team while recovering at home compared with 44% (33/75) of patients receiving only written discharge instructions (*P*=.02). Similar to patient satisfaction, age below or above the median did not alter reported connection scores in the texting group (*P*=.34). The type of procedure did influence the patient perception of connectedness. Patients in the texting group reported similar rates of connectedness regardless of type of procedure performed (*P*=.78). In contrast, those in the control group who underwent hysterectomy felt much more connected (22/37, 60%) compared with their counterparts undergoing uterine-sparing procedures (11/38, 29%; *P*=.008).

### Patient-Reported Value of Text Messaging

**Figure 3:**
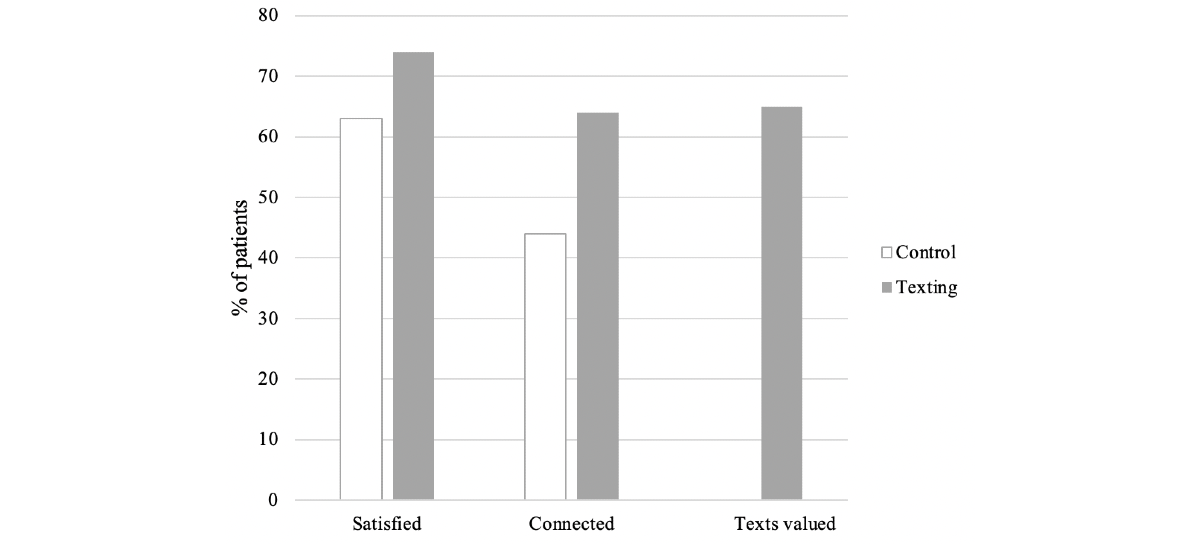
Percentage of patient with responses ≥9 on a scale of 1 to 10 to the following questions: “How connected did you feel to your healthcare team while recovering at home?” “How satisfied are you with your postoperative care?” “How valuable did you find the text messages while recovering at home?”.

The third and final question posed to the 62 survey respondents who received text messages during their postoperative survey was “How valuable did you find the text messages while recovering at home?” Of the patients receiving text messaging, 65% (40/62) reported value in the messaging (Figure 3).

## Discussion

### Principal Findings

Implementing a postoperative text messaging service is a feasible and potentially cost-effective way to deliver postoperative discharge instructions to patients undergoing laparoscopic benign gynecologic surgery. Text messaging was successfully received by 80% of intended intervention patients. This was accomplished at a cost of US $2.85 per patient. The study demonstrated patients receiving postoperative text messaging showed a trend toward an increase in satisfaction with their recovery and statistically significant increase in their sense of connection with their health care team. Largely, patients receiving text messaging found the texts valuable.

An unexpected finding was that patients undergoing uterine-sparing procedures had lower scores at baseline for satisfaction with recovery and connection with the health care team. These differences were eliminated with the text messaging intervention. A possible explanation may be the patients’ social support networks. Many women have undergone hysterectomies and are often willing to share advice on recovery, which might supplement the patient’s experience in recovery. Patients undergoing uterine-sparing procedures may not have as much access to this type of support. These findings may make the text message intervention even more valuable in a patient population undergoing uterine-sparing procedures.

Among patients selected to receive text messages, 20% did not receive the initial welcome text message, most likely due to use of a landline number instead of a mobile number and incorrect numbers in their charts. This error can be mitigated in a future study; however, this may limit eligible participants. The total cost for the text messages in this pilot study was US $178 to provide the full text message series to a total of 62 patients. It is difficult to quantify the financial benefit of increased patient satisfaction and connectedness. It is possible postoperative text messaging affords a significant return on investment through decreased complications requiring admission, decreased patient phone calls or messages, and decreased follow-up visits. Previous studies have demonstrated that the use of a mobile application for low-risk, postoperative, ambulatory patients led to deceased in-person follow-up visits [9]. The extension of this text messaging protocol to a larger number of patients may be cost-prohibitive if patients’ goodwill and a reduction in resource usage cannot be demonstrated. This study used a convenience sample of patients who received surgery over a 6-month period, so likely was not powered to detect statistically significant differences. A larger study that tracks complication rates and surveys office staff and providers is also necessary.

### Conclusions

Postoperative text messages proved to be feasible in a population of patients undergoing benign gynecologic laparoscopic procedures. Text messages demonstrated a trend toward increased patient satisfaction with recovery and statistically significant increase in perception of connection with the health care team. The trend was more pronounced in patients undergoing uterine-sparing procedures. Minimal errors in messaging were identified. Given increasing emphasis on patient experience and the practice of cost-effective health care, further evaluation of a postoperative text messaging protocol that is adequately powered is warranted to determine patient and resource allocation benefit.
